# 2-Pyrrolidinone and Succinimide as Clinical Screening Biomarkers for GABA-Transaminase Deficiency: Anti-seizure Medications Impact Accurate Diagnosis

**DOI:** 10.3389/fnins.2019.00394

**Published:** 2019-05-08

**Authors:** Adam D. Kennedy, Kirk L. Pappan, Taraka Donti, Mauricio R. Delgado, Marwan Shinawi, Toni S. Pearson, Seema R. Lalani, William J. Craigen, V. Reid Sutton, Anne M. Evans, Qin Sun, Lisa T. Emrick, Sarah H. Elsea

**Affiliations:** ^1^Metabolon, Inc., Morrisville, NC, United States; ^2^Department of Molecular and Human Genetics, Baylor College of Medicine, Houston, TX, United States; ^3^Department of Neurology and Neurotherapeutics, Texas Scottish Rite Hospital for Children, The University of Texas Southwestern Medical Center, Dallas, TX, United States; ^4^Department of Pediatrics, Washington University School of Medicine St. Louis, St. Louis, MO, United States; ^5^Department of Neurology, Washington University School of Medicine St. Louis, St. Louis, MO, United States; ^6^Department of Neurology, Baylor College of Medicine, Houston, TX, United States

**Keywords:** 2-pyrrolidinone, vigabatrin, GABA, neurometabolic, inborn error of metabolism, neurotransmitter, 4-aminobutyrate aminotransferase deficiency, GABA-transaminase deficiency

## Abstract

Broad-scale untargeted biochemical phenotyping is a technology that supplements widely accepted assays, such as organic acid, amino acid, and acylcarnitine analyses typically utilized for the diagnosis of inborn errors of metabolism. In this study, we investigate the analyte changes associated with 4-aminobutyrate aminotransferase (ABAT, GABA transaminase) deficiency and treatments that affect GABA metabolism. GABA-transaminase deficiency is a rare neurodevelopmental and neurometabolic disorder caused by mutations in *ABAT* and resulting in accumulation of GABA in the cerebrospinal fluid (CSF). For that reason, measurement of GABA in CSF is currently the primary approach to diagnosis. GABA-transaminase deficiency results in severe developmental delay with intellectual disability, seizures, and movement disorder, and is often associated with death in childhood. Using an untargeted metabolomics platform, we analyzed EDTA plasma, urine, and CSF specimens from four individuals with GABA-transaminase deficiency to identify biomarkers by comparing the biochemical profile of individual patient samples to a pediatric-centric population cohort. Metabolomic analyses of over 1,000 clinical plasma samples revealed a rich source of biochemical information. Three out of four patients showed significantly elevated levels of the molecule 2-pyrrolidinone (*Z*-score ≥ 2) in plasma, and whole exome sequencing revealed variants of uncertain significance in *ABAT*. Additionally, these same patients also had elevated levels of succinimide or its ring-opened form, succinamic acid, in plasma, urine, and CSF and/or homocarnosine in urine and CSF. In the analysis of clinical EDTA plasma samples, the levels of succinamic acid and 2-pyrrolidinone showed a high level of correlation (*R* = 0.72), indicating impairment in GABA metabolism and further supporting the association with GABA-transaminase deficiency and the pathogenicity of the *ABAT* variants. Further analysis of metabolomic data across our patient population revealed the association of elevated levels of 2-pyrrolidinone with administration of vigabatrin, a commonly used anti-seizure medication and a known inhibitor of GABA-transaminase. These data indicate that anti-seizure medications may alter the biochemical and metabolomic data, potentially impacting the interpretation and diagnosis for the patient. Further, these data demonstrate the power of combining broad scale genotyping and phenotyping technologies to diagnose inherited neurometabolic disorders and support the use of metabolic phenotyping of plasma to screen for GABA-transaminase deficiency.

## Introduction

**FIGURE 1:**
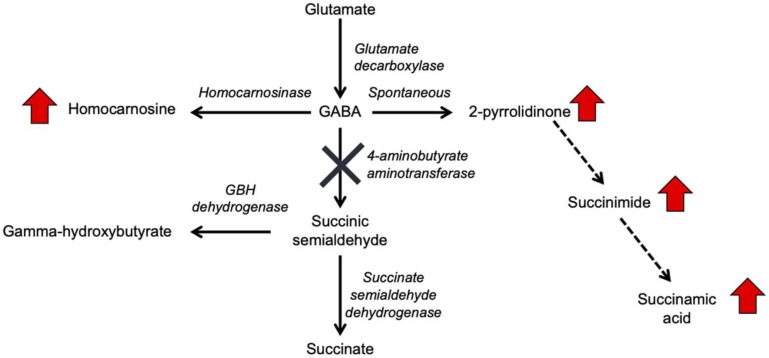
GABA metabolism pathways are altered due to GABA-transaminase deficiency and treatment affecting GABA metabolism. The entire pathway from glutamate conversion to GABA through succinate formation is represented along with the respective enzymes for each step. Due to tissue-specific expression of the enzymes, not all molecules are detected in each biological matrix (e.g., homocarnosine is present below the limit of detection in plasma).

Gamma-aminobutyric acid (GABA) transaminase deficiency (OMIM #613163), also known as 4-ABAT deficiency is a rare genetic disorder that disrupts the degradation of GABA to succinic semialdehyde (Figure 1). This IEM is due to decreased or deficient activity of the enzyme 4-ABAT (ABAT, EC 2.6.1.19, OMIM #137150). GABA-transaminase deficiency is an ultra-rare disease reported and characterized in only a few patients (Medina-Kauwe et al., 1998, 1999; Tsuji et al., 2010; Besse et al., 2016; Louro et al., 2016; Koenig et al., 2017; Nagappa et al., 2017). The few reports suggest it is much rarer than other disorders of GABA metabolism, including glutamate decarboxylase deficiency (OMIM 603513), SSADH deficiency (OMIM 271980), and homocarnosinosis (serum carnosinase deficiency) (OMIM 236130) (Jakobs et al., 1993).

GABA has a relatively short half-life in plasma, is rapidly absorbed, and has both endocrine and hormonal effects (Abel and McCandless, 1992; Li et al., 2015; Maguire et al., 2015). GABA serves as the primary inhibitory neurotransmitter in the human nervous system, and GABA metabolism to succinic semialdehyde helps to regulate its levels and neurotransmitter activity. The accumulation of GABA, either through enzymatic inactivity of ABAT or medical intervention, can result in elevated levels of 2-pyrrolidinone, due to cyclization of GABA (Callery et al., 1978). Conversely, 2-pyrrolidinone can be converted to GABA when it is administered intravenously (Callery et al., 1979) or orally (Fasolato et al., 1988). 2-pyrrolidinone can be converted to succinimide through a two-step reaction (Bandle et al., 1984), and hydrolytic ring opening of cyclic imides such as succinimide can occur through enzymatic (Maguire and Dudley, 1978) and non-enzymatic (Kurono et al., 2008; Lerner et al., 2013) routes.

Aminobutyrate aminotransferase catalyzes the conversion of GABA to succinic semialdehyde, which is then oxidized to succinate by SSADH. Succinate can enter the tricarboxylic acid cycle for NADH and FADH_2_ production. Inhibitors of ABAT include valproate (Li et al., 2016; Piplani et al., 2016), vigabatrin (Petroff et al., 1995, 1996a,b), and topiramate (Meldrum and Rogawski, 2007; Porter et al., 2012). The inhibition of ABAT activity results in the accumulation of GABA, β-alanine, homocarnosine, and 2-pyrrolidinone (Jaeken et al., 1990; Parviz et al., 2014). Increased levels of GABA lead to symptomatic features of GABA-transaminase deficiency, including psychomotor retardation, hypotonia, hyperreflexia, lethargy, refractory seizures, abnormal brain magnetic resonance imaging (MRI) and electroencephalogram (EEG) abnormalities (Pearl et al., 2009; Dracopoulos et al., 2010; Schonstedt et al., 2015; Hussain et al., 2017). Traditional diagnostic avenues for GABA-transaminase deficiency include enzymatic testing of ABAT, neurotransmitter profiling of CSF – including GABA, and molecular testing of *ABAT* for pathogenic variants. Biomarkers of GABA-transaminase deficiency include elevated GABA in CSF; however, CSF GABA may not be included in some clinically available neurotransmitters analyses, limiting the diagnostic utility.

As a neurometabolic disorder, GABA-transaminase deficiency typically presents initially with hypotonia and may present with difficult to control seizures, including infantile spasms. Seizures can sometimes be moderated through dietary and/or medical intervention such as ketogenic diets which can influence seizure activity and GABA levels (Dahlin et al., 2005; Bough, 2008; Nylen et al., 2008; Yudkoff et al., 2008; Suzuki et al., 2009; Woolf et al., 2015; Li et al., 2017). Topiramate and vigabatrin are anti-seizure medications utilized to treat seizure disorders including infantile spasms. Vigabatrin is an irreversible inhibitor of ABAT that causes increased levels of GABA in the brain (Gram et al., 1989; Sheean et al., 1992; Petroff et al., 1999a; Kang et al., 2003; Rogawski and Loscher, 2004). In a rat model, inhibition of ABAT with vigabatrin resulted in decreased ABAT activity in brain, liver, and kidney and significantly increased levels of GABA in liver, brain, and plasma (Qume and Fowler, 1996). Reversible changes on brain MRI including T2 hyperintensities in the basal ganglia, brainstem and dentate nucleus have been reported in patients taking vigabatrin for infantile spasms (Pearl et al., 2009). The exact mechanism of action of topiramate is not known; however, inhibition of the binding of GABA to GABA-A receptors, resulting in increased levels of brain GABA, homocarnosine, and 2-pyrrolidinone (Petroff et al., 1999b) has been proposed (Meldrum and Rogawski, 2007).

Untargeted MS, also known as clinical metabolomics, can identify compounds routinely tested in patients diagnosed with IEMs, as well as analytes for which no clinical testing is available in the United States (Miller et al., 2015; Kennedy et al., 2016, 2017). However, treatment with the medications topiramate or vigabatrin also raises plasma 2-pyrrolidinone levels. While the similarity in metabolic phenotype could interfere with the utility of this biomarker in the detection of GABA-transaminase deficiency, drug treatment can be discerned from patient records (and metabolomic results), and the elevation of 2-pyrrolidinone – whether due to GABA-transaminase deficiency or anti-seizure medication – clearly separates these cases from the vast majority of other clinical cases tested. Here, we describe a broader metabolic analysis of GABA metabolites and demonstrate that the pattern of metabolite levels can be used to distinguish GABA-transaminase deficiency from treatment with topiramate and valproate that inhibit GABA-transaminase, improving the ability to accurately screen and diagnose GABA-transaminase deficiency.

## Materials and Methods

### Sample Collection

All procedures were performed in accordance with the ethical standards of the United States Department of Health and Human Services and were approved by the Baylor College of Medicine and Washington University Institutional Review Boards. All subjects or their parents/guardians gave written informed consent for publication in accordance with the Declaration of Helsinki. Specimens used in this analysis were referred for clinical metabolomic testing to our clinical biochemical genetics laboratory. All plasma samples were isolated from whole blood collected in EDTA-containing tubes at the site of collection and frozen. All EDTA plasma, urine, and CSF clinical samples were stored at −20°C until analyzed.

### Patients

Patients with a diagnosis of GABA-transaminase deficiency were identified either as part of this analysis (Patients 3 and 4) or were previously identified and reported (Patients 1 and 2) (Besse et al., 2015, 2016; Koenig et al., 2017).

### Case Presentations

#### Patient 1

**FIGURE 2:**
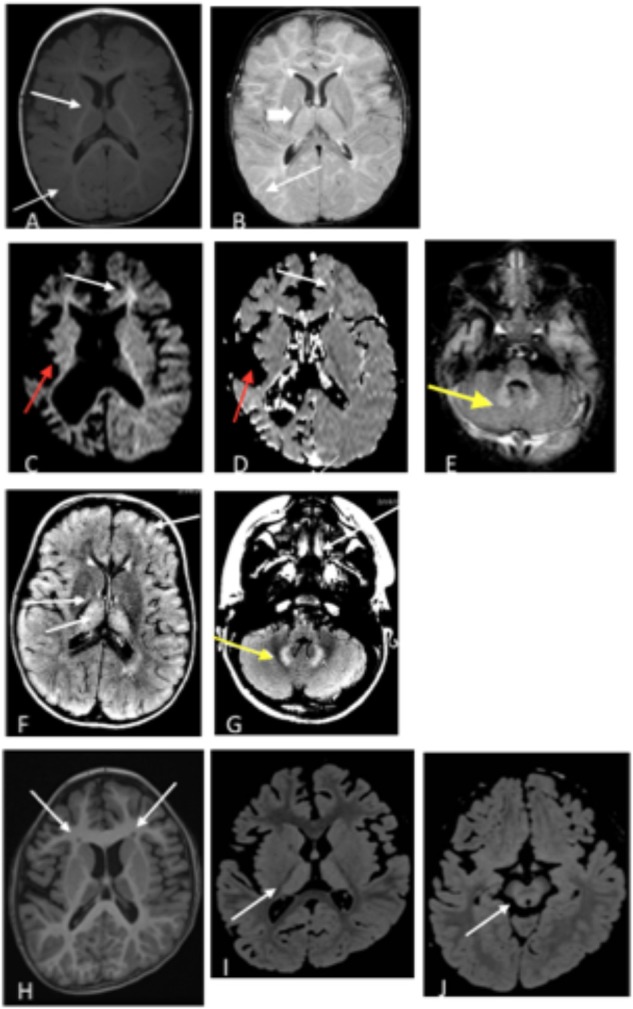
**(A,B)** Patient 1 at 9 months old. **(A)** T1 axial image and **(B)** T2 axial image. No evidence of atrophy or injury. White arrows demarcate areas of delayed myelination; thick arrow show abnormal hyperintensity in bilateral thalamus. Images **(C–E)**. Patient 2 at 17 months old. **(C)** Axial diffusion weighted imaging (DWI) and **(D)** Axial apparent diffusion coefficient (ADC). Severe bilateral cerebral atrophy right side (red arrows) greater than the left side. Enlarged lateral ventricles secondary to ex vacuo loss. White arrows demonstrate acute restricted diffusion in white matter than can be related to acute injury. **(E)** Axial T2 FLAIR image with T2 hyperintensities indicated by yellow arrow in bilateral dentate nuclei. **(F,G)** Patient 3, age 3 years. T2 FLAIR axial images are shown. **(F)** White arrow shows T2 hyperintensities in bilateral thalami, and **(G)** Yellow arrow shows T2 hyperintensities in dentate nucleus. **(H–J)** Patient 4 at age 6 years. **(H)** Axial T1 image shows bilateral frontal periventricular heterotopia (white arrows). **(I,J)** Axial FLAIR images showed patchy hyperintensity in the thalamus **(I)** and midbrain **(J)** (white arrows).

A 21-month old male presented with hypotonia and global developmental delay (Besse et al., 2016; Koenig et al., 2017). At 21 months, he sat supported, was non-verbal but understood “no” and could wave as a gesture to communicate goodbye. He had intermittent upward eye deviation, diagnosed as oculomotor apraxia, head drops, hand twitching, and continued episodes of staring. Continuous EEG monitoring was abnormal with a slow background and multi-focal spike and wave but without electrographic changes with the abnormal movements. He had delayed myelination on brain MRI at 9 months old with mild increased T2 signal in the bilateral thalamus (Figures 2A,B). CSF studies for neurotransmitters (excluding GABA) and amino acids were normal. Whole exome sequencing revealed compound heterozygous variants of uncertain significance (VUS) inherited in *trans* in *ABAT* (c.454C > T, p.P152S and c.1393G > C, p.G465R). Diagnosis was confirmed with CSF GABA elevated at 247 nmol/L (normal range 17–67 nmol/L).

#### Patient 2

Patient 2 is a 6-year old male (at the time of testing) previously reported with GABA-transaminase (Besse et al., 2015). He presented initially with hypotonia and vision impairment. He had severe progressive psychomotor retardation with inability to even hold his head, non-verbal, medically refractory intractable seizures, and cortical visual impairment. His initial EEG was normal early in infancy but progressed with generalized slowing and multifocal spike and wave activity. MRI at age 17 months reported severe global atrophy involving the right cerebral hemisphere more than left and signal abnormalities involving bilateral internal capsules and dentate nuclei and decreased amount of white matter, as well as delayed myelination (Figures 2C–E). He had similarly affected sisters who died at age 9 years and 1 year old. Whole exome sequencing revealed a homozygous VUS in *ABAT*, c.631C > T (p.L211F), later shown to result in enzyme deficiency (Besse et al., 2015).

#### Patient 3

Patient 3 is a 4-year old male, born by C-section due to fetal macrosomia. Evaluation at 4 years of age revealed motor delays (walked independently at 3 years of age) and speech delays (20–30 words), mild hypotonia, significantly ataxic gate (frequent falls), autistic features (behavioral outbursts in unfamiliar settings, hypersensitive to noise and aversion to anything touching his head) and strabismus. Brain MRI revealed abnormal increased signal in the T2 imaging in thalami, brainstem, globus pallidus, and cerebellar dentate nuclei bilaterally, as well as in deep and subcortical white matter (Figures 2F,G). No seizures have been reported to this date. Whole exome sequencing identified two variants inherited in *trans* in *ABAT:* a c.168+1G > A likely pathogenic variant and heterozygous c.638T > G (p.F213C) VUS. No other significant findings were present in this patient at the time of evaluation.

#### Patient 4

Patient 4 is a 7-year-old female who initially presented at age 5 months with hypotonia, failure to thrive, and global developmental delay. Generalized chorea, characterized by writhing movements of all limbs and tongue thrusting, emerged between 6 and 12 months. She also developed multifocal jerky movements suggestive of myoclonus. Her movement disorder is partially controlled with clonazepam and levetiracetam. She has profound developmental delay, with absent head control and a general paucity of purposeful voluntary movements. There is no history of clinical seizures. EEG at age 3 years demonstrated generalized slowing, and a repeat study at 7 years showed multifocal sharp waves. Brain MRI at age 6 years demonstrated frontally predominant cerebral atrophy, bilateral frontal periventricular nodular heterotopia, thick corpus callosum, and abnormal areas of T2 hyperintensity in bilateral thalami and midbrain (Figures 2H–J). The patient is the product of a close consanguineous union. Whole exome sequencing detected a homozygous VUS in *ABAT*, c.1394G > A, p.G465D. In addition, the patient is homozygous for a VUS in *LRRC7*, c.2938C > T, p.R980X. *LRRC7* has not been associated with a human disease. Diagnosis of GABA-transaminase deficiency was confirmed with significantly elevated levels of free and total GABA in CSF: 272 (nM) and 32.2 (μM), respectively (reference ranges: free GABA, 32–170 nM and total GABA, 3.3–12.2 μM) (Baylor Institute of Metabolic Disease, Dallas).

### Biochemical Profiling

Metabolomic profiling was performed by Baylor Genetics Laboratories (Houston, TX, United States) and Metabolon, Inc., (Morrisville, NC, United States), as described previously (Evans et al., 2009, 2014; Miller et al., 2015) for plasma, urine (Kennedy et al., 2016), and CSF (Kennedy et al., 2017) using two different metabolomics platform configurations. For both configurations, small molecules were extracted from 100 μl of sample in an 80% methanol solution.

The first platform configuration (platform version 1) consisted of four analyses: GC-MS, LC-MS/MS in positive mode (LCMS Pos), LC-MS/MS in negative mode (LCMS Neg), and a LC-MS/MS Polar method (LCMS Pol). GC-MS was performed on bistrimethyl-silyl-trifluoroacetamide derivatized analytes using a Trace DSQ fast-scanning single-quadrupole mass spectrometer (Thermo Finnigan). For LC/MS Neg and LCMS Pos methods, chromatographic separation was completed using an ACQUITY UPLC (Waters) equipped with a Waters BEH C18 column followed by analysis with an Orbitrap Elite high-resolution mass spectrometer (Thermo Finnigan).

The second configuration (platform version 2) utilized the following chromatographic methods: LCMS Neg (same as version 1), LCMS Pol (same as version 1), and LCMS positive ion method focusing on lipophilic compounds (LCMS Pos Lipid), and LCMS positive ion method focusing on polar compounds (LCMS Pos Polar).

Comparison of the results of the analytical performance of the two versions of the platforms showed equivalent performance based on molecules detected on the arms of the platform, intra- and inter-day precision, linearity, limit of detection, and comparison to currently utilized methods. Additionally, the reference populations and anchor matrix samples showed equivalence as measured by the *Z*-score reference population analysis. Reference cohorts for EDTA plasma, urine, and CSF were developed from residual normal samples remaining from pediatric patients referred to the Baylor College of Medicine Biochemical Genetics Laboratory for metabolic screening. For platform version 1, 190 EDTA plasma samples were profiled and covered 725 biochemicals of known identity (named compounds; Miller et al., 2015), 100 urine samples were profiled and detected 663 named compounds (Kennedy et al., 2016), and CSF from 100 samples were analyzed with the detection of 449 named compounds. For platform version 2, the EDTA plasma reference cohort was comprised of 536 pediatric samples and spanned 701 named compounds, and the CSF cohort (90% pediatric) contained 79 samples in which 426 named biochemicals were covered.

Each run day of clinical samples for analysis included four technical replicates of anchor samples. These anchor samples consisted a pool of plasma, urine, or CSF samples from over 100 donors each. The anchors were utilized to median normalize each run day to compare clinical samples directly across different run days and to the reference population database.

In both configurations, Tier 1 (Sumner et al., 2007) metabolite identification was achieved by matching ion chromatographic retention index, accurate mass, and mass spectral fragmentation signatures with those of known chemical structures in a reference library created from authentic standards measured by the identical analytical procedure (DeHaven et al., 2010).

### Statistical Analysis

Semi-quantitative analysis was achieved by comparing patient samples to a set of invariant anchor specimens included in each batch. Raw spectral intensity values for each biochemical were median normalized to the anchor samples, log transformed, and compared to a normal reference population, which consisted of more than 1,000 unique samples, to generate *Z*-score values. Rare compounds are those analytes detected in the patient specimen but only rarely seen in the reference population (<10% of all patients tested). Median raw intensity values were calculated for all analytes identified in ≥2/3 of the anchor specimens and these median values were then used to normalize corresponding analyte raw intensity values in the patient specimen. Analytes not identified in at least 2/3 of the anchor specimens were excluded from analysis. Data collected from urine samples were normalized to creatinine. *Z*-scores were calculated using the mean and standard deviation of the entire median-scaled log-transformed dataset.

To compare the number of biochemicals in ABAT and non-ABAT groups, Welch’s two-sample *t*-tests were used to identify differences in numbers of biochemicals capable of distinguishing between the GABA-transaminase deficiency and non-ABAT-related cases on GABA-raising medications where *p* < 0.05 was considered statistically significant.

## Results

Global biochemical profiling of EDTA plasma was performed on six samples from four unique subjects with GABA-transaminase deficiency (Table 1) and several subjects without variants in *ABAT* taking medication that inhibits GABA-transaminase or otherwise may alter GABA metabolism (*n* = 93). Urine from two and CSF from three of the subjects were also analyzed by global biochemical profiling (Table 1). A total of 566 ± 40 compounds was detected in ABAT EDTA plasma samples (*n* = 6) versus 572 ± 32 in non-ABAT samples (*n* = 1,088, not statistically significant different, *p* > 0.05; Supplementary Data Sheet S1). The average plasma 2-pyrrolidinone level was elevated by over 4.34 standard deviations (range 2.74–6.88) in subjects with GABA-transaminase deficiency (as reported by *Z*-scores) relative to a normal reference population (Table 2 and Supplementary Data Sheet S1).

**TABLE 1:** Clinical demographics of patients diagnosed with GABA-transaminase deficiency.

**Patient**	**Sample ID^1,2^**	**Sample** **type^2^**	**Age^∗^**	**Gender**	**Ethnicity**	***ABAT* variants^+^** **clinical report**	**Genome (hg38)** **Chromosome 16**	**ClinGen canonical ID**	**Medications**	**Diet**
1	*CSF01A* *P01A* *P01B* *U01A* **P01C**	CSF EDTA Plasma EDTA Plasma Urine **EDTA** **Plasma**	15 mon – 2 y	Male	Hispanic	c.454C>T (p.Pro152Ser) c.1393G>C (p.Gly465Arg) VUS, *in trans*	g.8764744C>T g.8781320G>C	CA394688322 CA394691458	Milk of Magnesia, Prevacid, Omega-3, Lansoprazole	Low glutamate
2	*U02A* **P02A**	Urine **EDTA** **Plasma**	6 y	Male	Hispanic	c.631C>T (p.Leu211Phe) homozygous	g.8768220C>T	CA175085	Thiamine, Levocarnitine, Coenzyme Q10, Keppra, Levetiracetam, Clonazepams	G-button feeds
3	***CSF03A*** **P03A**	**CSF** **EDTA** **Plasma**	4 y	Male	Caucasian	c.168+1G>A, likely pathogenic variant c.638T>G (p.Phe213Cys), heterozygous VUS, *in trans*	g.8746099G>A g.8768227T>G	CA394692408 CA394688780	No medications	No special diet
4	**P04A** **CSF04A**	**EDTA** **Plasma** **CSF**	6 y	Female	Caucasian	c.1394G>A (p.Gly465Asp), VUS, homozygous^3^	g.8781321G>A	CA16607451	Miralax, Albuterol, Keppra, Clonazepam	G-tube feeds with Pediasure

*^1^Samples in italics were analyzed on Platform version 1. All other samples were analyzed on Platform version 2. ^2^Succinamic acid was measured on the Polar arm of Platform 2 in samples in boldface. ^3^Other significant WES variant identified, *LRRC7*: c.2938C>T (p.R980X), homozygous. ^+^P80404, ENST00000396600. ^∗^y, years; mon, months.*

**TABLE 2:** Metabolomics identifies altered levels of molecules connected to GABA metabolism in GABA-transaminase deficiency patients*.

**Patient**	**Sample**	**Matrix**	**2-pyrrolidinone**	**Succinimide**	**Succinamic Acid^1^**	**Glutamate**	**GABA**	**Succinate**	**Homocarnosine**
1	*CSF01*A	CSF	7.05	5.76	NA	−1.54	0.15	1.65	2.60
	*U01A*	Urine	3.77	4.94	NA	−0.21	0.96	0.21	1.56
	*P01A*	EDTA plasma	6.16	ND	NA	0.71	ND	0.73	ND
	*P01B*	EDTA plasma	6.88	ND	NA	0.86	ND	−0.44	ND
	**P01C**	**EDTA plasma**	4.73	Significant rare^##^	**3.83**	0.03	ND	0.40	ND
2	*U02A*	Urine	0.69	1.55	NA	−0.62	0.92	0.63	0.87
	**P02A**	**EDTA plasma**	1.92	Significant rare^##^	**0.62**	0.92	ND	0.77	ND
3	**P03A**	**EDTA plasma**	2.19	Significant rare^##^	**1.80**	0.57	ND	1.65	ND
	***CSF03A***	**CSF**	5.18	Significant rare^##^	**1.57**	−0.61	0.31	−1.28	1.91
4	**P04A**	**EDTA plasma**	3.58	Significant rare^##^	**2.02**	−0.78	ND	−0.51	ND
	**CSF04A**	**CSF**	Significant rare^#^	Significant rare^##^	**2.25**	−1.15	ND	−1.51	2.75
Non-GABA-T vigabatrin-treated	***N* = 12**	**EDTA Plasma**	2.88+/−1.41	NA	1.88+/−1.19	0.15+/−0.90	ND	−1.05+/−2.16	ND

*Z-scores are shown. ND, not detected; NA, not applicable. ^1^Z-Scores for succinamic acid were calculated for samples in boldface. ^#^Reference ranges were not definable for 2-pyrrolidinone in CSF on platform version 2. 2-pyrollidinone is considered a rare molecule on this platform due to limited detection in the reference population; raw values are considered in the interpretation of this finding. ^##^Reference ranges were not definable for succinimide in EDTA Plasma or CSF on platform version 2. Succinimide is considered a rare molecule on this platform due to limited detection in the reference population; raw values are considered in the interpretation of this finding.

**FIGURE 3:**
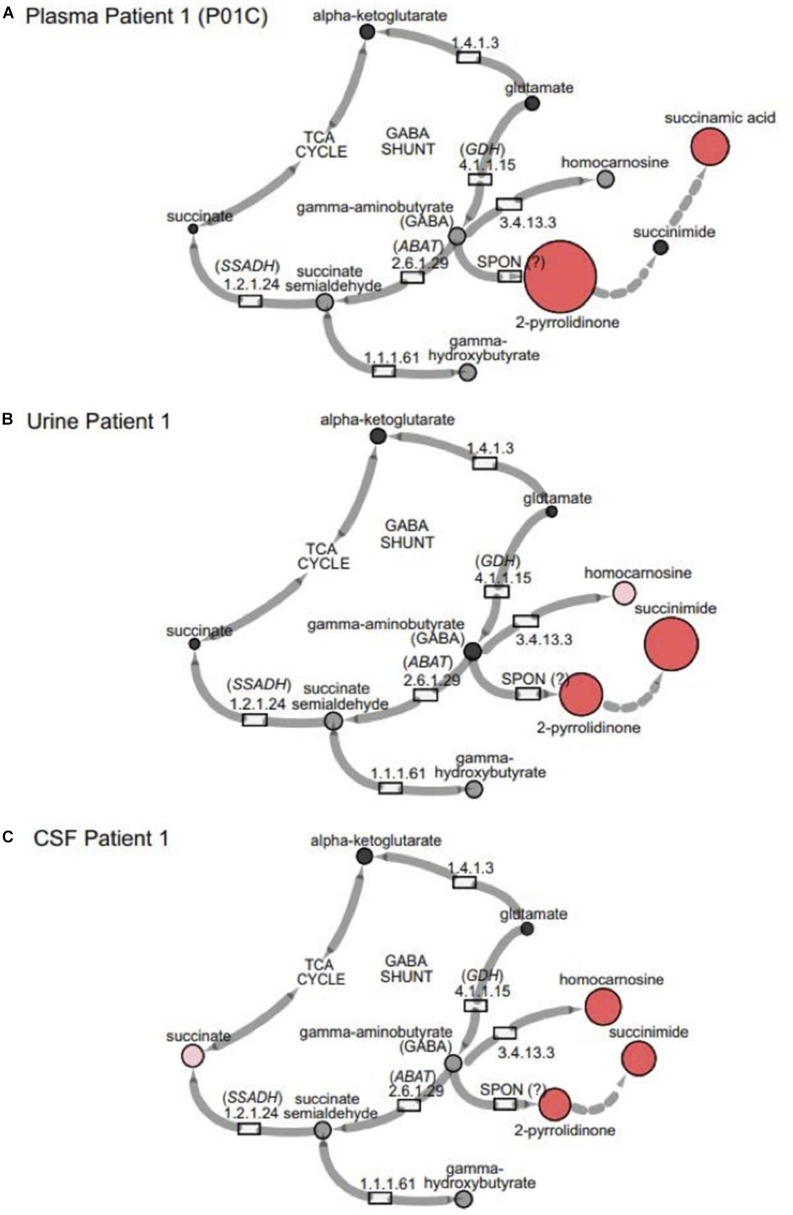
GABA metabolites are altered in GABA-transaminase deficiency and in use of treatments affecting GABA metabolism. Representative pathway images are shown for GABA-transaminase deficiency Patient 1 in **(A)** EDTA Plasma, **(B)** Urine, and **(C)** CSF. Each image shows the relative accumulation of biochemicals (red circles) or trending increases (pink circles, 1.5 ≤ *Z* < 2). The size of each of the circles is representative of the *Z*-score for that biochemical. Black circles represent molecules with *Z*-scores between –1.5 and 1.5 (–1.5 < *Z* < 1.5) or detected rare molecules for which a *Z*-score could not be calculated. Gray circles represent biochemicals in the library but not detected in the samples using Cytoscape to delineate biochemical pathways (http://cytoscape.org) (Shannon et al., 2003). All enzymes in the pathway are denoted by their EC designations. *GDH*, glutamate dehydrogenase; *SSADH*, succinic semialdehyde dehydrogenase; *ABAT*, aminobutyrate aminotransferase; SPON, spontaneous.

**FIGURE 4:**
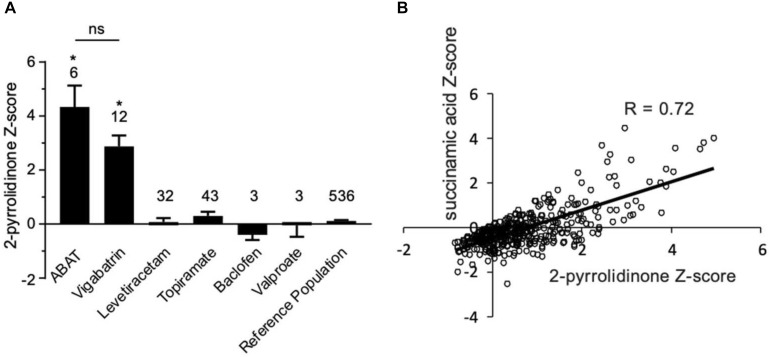
Box plot profile for 2-pyrrolidinone levels detected in plasma for all clinical samples. **(A)** *Z*-scores for all clinical EDTA plasma samples are plotted using a box plot format. ABAT cases show elevated *Z*-scores for 2-pyrrolidinone versus those patients without GABA-transaminase deficiency and treated with vigabatrin, topiramate, and/or valproate. If patients were receiving vigabatrin in addition to other treatments, they were grouped with vigabatrin. Bars represent the mean +/– the standard error of the mean (SEM) of the *Z*-scores for 2-pyrrolidinone for each group. The numbers above the bars represent the number of unique patient samples for each group. *p < 0.05. NS indicates a comparison which was not statically significant, *p* > 0.05. **(B)** Correlation of 2-pyrolidinone levels to succinamic acid levels in clinical plasma samples. Samples which had both 2-pyrrolidinone and succinamic acid *Z*-scored (*n* = 409) are plotted showing a significant positive correlation with these two molecules.

In the case of GABA-transaminase deficiency, it would be expected that the molecules participating in reactions preceding the defective enzyme would accumulate to higher levels than normal/healthy levels. The pathway outlined in Figure 1 shows the enzymes involved in GABA metabolism and the position of GABA transaminase (ABAT) in this biological process. For Patient 1, we analyzed EDTA plasma, urine, and CSF. Biochemical profiling, performed at two independent time points in plasma, showed an elevated 2-pyrrolidinone with *Z*-scores of 6.16 and 6.88, respectively (Table 2 and Figures 3A, 4A). Similar findings for 2-pyrrolidinone were observed in both CSF (*Z* = 7.05) and urine (*Z* = 3.77) (Table 2 and Figures 3B,C). CSF showed a succinimide level of *Z* = 5.76, and the urine sample for Patient 1 had a succinimide level of *Z* = 4.94. For Patient 2, a urine sample was analyzed and showed 2-pyrrolidinone levels within the expected range of the reference population (*Z* = 0.69).

By the time additional samples from subsequent ABAT patients were acquired, the configuration of the metabolomics platform had matured. The description of the platform configuration is outlined in the Materials and Methods section, and Table 1 delineates which samples were run on the respective configurations of the platform. Four plasma samples, one from each of the four patients, were subsequently analyzed. 2-pyrrolidinone *Z*-scores ranged from 1.92 to 4.73 across these four EDTA plasma samples (Table 2). For the EDTA plasma sample from Patient 1, succinamic acid, or ring-opened succinimide, showed a relative elevation (*Z* = 3.83), and *Z*-scores for succinamic acid in EDTA plasma and CSF samples from the other GABA-transaminase deficiency patients ranged from 0.62 to 2.25 (Table 2). Succinimide was detected in all EDTA plasma and CSF samples from GABA-transaminase deficiency patients analyzed on platform 2 but was not detected in enough of the healthy reference control population samples to permit the calculation of *Z*-score reference ranges. Of all clinical EDTA plasma samples run on platform version 2, *Z*-scores were obtained for both succinamic acid and 2-pyrrolidinone in 409 samples. Correlation of the values within these samples for succinamic acid and 2-pyrrolidinone showed a high degree of correlation (*R* = 0.72, Figure 4B). The considerable correlation between 2-pyrrolidinone and succinamic acid is suggestive of a product-substrate relationship, even though the biological mechanism leading to the conversion of one to the other is only partially understood.

Some molecules, such as homocarnosine, are expressed in a tissue-specific manner (Sjaastad et al., 1976). Homocarnosine is a brain-specific dipeptide. It was not detected routinely in plasma, most likely due to its limit of detection but did show elevated levels in the CSF and urine of GABA-transaminase deficiency Patients 1 and 4 (Figure 3 and Table 2).

We have seen that 2-pyrrolidinone manifested elevated levels in patients other than those diagnosed with GABA-transaminase deficiency. Several pharmacological interventions for seizures were identified as part of the biochemical profile. Vigabatrin, topiramate, levetiracetam, baclofen, and valproate treatment were identified through the detection of the drugs themselves or by metabolites of the drug intervention, e.g., 3-hydroxyvalproate for valproate treatment. The average *Z*-scores for 2-pyrrolidinone in patients with the different medications were 2.87, 0.07, 0.30, −0.39, and −0.05 for those patients treated with vigabatrin, levetiracetam, topiramate, baclofen, and valproate, respectively (Figure 4A, Table 2, and Supplementary Data Sheet S2). The average 2-pyrrolidinone *Z*-score for the samples from patients diagnosed with GABA-transaminase deficiency was 4.24 (Figure 4A; Table 2). The levels of 2-pyrrolidinone were significantly higher in patients with GABA-transaminase deficiency and in patients treated with vigabatrin versus the reference population (*p* < 0.05). The levels of 2-pyrrolidione were not different at a statistically significant level between patients with GABA-transaminase deficiency and patients treated with vigabatrin (*p* = 0.1043).

## Discussion

Biochemical phenotyping, the systematic study of small-molecule products of biochemical pathways, has been used to predict, diagnose and monitor the effects of treatment of inborn errors of metabolism (Atwal et al., 2015; Miller et al., 2015, 2016; Burrage et al., 2016; Donti et al., 2016; Kennedy et al., 2016). We applied an untargeted metabolomics profiling approach on plasma, urine, and CSF from patients with known or suspected GABA-transaminase deficiency and non-*ABAT*-related patients treated with medications that inhibit GABA metabolism with the objective of unraveling metabolic biomarkers to differentiate disorder from treatment (Figures 3, 4). As with the diagnostic and clinical utility of another neurometabolic disorder, aromatic amino acid decarboxylase deficiency (Atwal et al., 2015), the analysis of plasma (venipuncture) or urine is less invasive than a lumbar puncture, less expensive, and can be performed faster than CSF neurotransmitter analysis or DNA testing. Collection of an appropriate and necessary volume of CSF can be difficult, and rigorous sample handling as well as storage protocols are required to preserve biomarkers appropriately. 2-pyrrolidinone is an indicator of shunted GABA metabolism, which can be affected either through inhibited ABAT activity or through medical intervention such as topiramate or vigabatrin which alters GABA metabolism (vigabatrin) or GABA biological activity (topiramate).

Vigabatrin is utilized to treat seizures in SSADH deficiency, another IEM of GABA metabolism which presents with many of the same clinical features as GABA-transaminase deficiency [reviewed by Vogel et al. (2013)]. Subjects with SSADH deficiency have accumulated levels of γ-hydroxybutyric acid (GHB) in their bloodstream and tissues and vigabatrin treatment lowers GHB levels through GABA transaminase inhibition (Pearl and Gropman, 2004). This inhibition is most significant in the brain as vigabatrin has little to no effect on peripheral GABA transaminase (Pearl et al., 1993). In relation to other IEMs, vigabatrin treatment also down-regulates the transcription of aromatic amino acid decarboxylase (Buckland et al., 1996) and may drive increased levels of 3-methoxytyrosine (Atwal et al., 2015). In our cases, vigabatrin did not significantly alter 3-methoxytyrosine levels, but testing additional samples may help characterize how vigabatrin treatment affects 3-methoxytyrosine levels in plasma, urine, or CSF. Therefore, analyzing multiple biomarkers at a time could differentiate true disease signatures affecting disparate pathways versus those biomarker signatures resultant through treatment. In this study, the identification of medications alongside the levels of 2-pyrrolidinone gave insight into the mechanism of the elevation of 2-pyrolidinone as a clinical biomarker (Figure 4). Further supporting the association of inhibition of GABA transaminase in the pathology of this condition, the patients with GABA-transaminase deficiency all of have some findings on imaging previously reported in patients with vigabatrin toxicity (Figure 2). Patients 1, 3 and 4 have bilateral T2 hyperintensities in the thalami. Patients 2 and 3 have bilateral T2 hyperintensities in the dentate nucleus, and Patient 4 also has involvement of the brainstem, offering yet an additional route to diagnosis of GABA-transaminase deficiency.

Metabolic biomarkers of disease accumulate to significantly increased levels at points in pathways prior to the defective enzymatic step. Consistent with this phenomenon, biochemicals downstream of the affected enzyme decrease in abundance. However, some biochemicals can be synthesized through multiple mechanisms. For example, succinate can be formed through amino acid catabolism, TCA cycle anaplerosis, and oxidation of odd-chain fatty acids. Additionally, some mutations in *ABAT* can express residual enzymatic activity and not a complete ablation of enzymatic activity resulting in “partial” decreases in metabolites downstream of ABAT (Pop et al., 2015). As succinate also is formed downstream of ABAT activity, and it is involved in mitochondrial physiology, a diagnosis of GABA-transaminase deficiency needs to be differentiated from mitochondrial diseases due to mutations in mitochondrial DNA (Besse et al., 2015) or other aspects of mitochondrial physiology (Ravasz et al., 2017). Building panels of biomarkers specific for an increasing number of diverse diseases can help to further increase the utility of biochemical phenotyping as a screening technology.

Biochemical phenotyping can differentiate signatures of disease from those signatures of treatment associated with the same biochemical pathway. Gathering additional samples from other metabolic disorders and different therapeutic interventions will help to strengthen this technology. Mutations in the genes for GDH and succinic SSADH are associated with respective IEMs. GDH deficiency can be responsive to vitamin B_6_ therapy (Villela and Calcagnotto, 1977a, b). Early detection of disease can result in expedited treatment, and this technology and assay can then track effects of treatment (e.g., alleviation of disease signatures) or track a how patients may respond to treatment (e.g., differentiate responders from non-responders). In the analysis of over 1,000 clinical samples, we identified and confirmed two new cases of GABA-transaminase deficiency (Patients 3 and 4), in addition to two previously described cases (Patients 1 and 2). The patients presented exhibited phenotypic variability with all having hypotonia, abnormal movements, and eventually abnormal EEGs, but not all had seizures.

This broad screening tool can provide functional genomic data to assess the pathogenicity of VUS and identify the biomarkers of disease and, in the case of 2-pyrrolidinone, identify cases where GABA metabolism is perturbed due to disease (GABA-transaminase deficiency) or by pharmaceutical treatment that affects GABA metabolism. While treatments which elevate 2-pyrrolidinone levels through GABA-transaminase inhibition can interfere with the use of 2-pyrrolidinone as a biomarker for screening GABA-transaminase deficiency, the treatment of patients with these drugs can be discerned from the patient medical history, and many of these medications can also be detected in clinical metabolomic analysis of plasma. As mentioned in a preliminary case study citing clinical data and interpretations from our clinical diagnostic platform (Koenig and Bonnen, 2018), monitoring 2-pyrrolidinone and levels by metabolomic profiling in patients with GABA-transaminase deficiency may inform the efficacy of therapeutic intervention. Here, we show that the integrated use of metabolomic and genomic testing provides an informed approach to diagnose GABA-transaminase deficiency and distinguish it from other sources of GABA/2-pyrrolidinone elevations, that may be otherwise confounding in targeted GABA analyses. These data demonstrate the power of combining broad scale genotyping and phenotyping technologies to diagnose inherited neurometabolic disorders and indicate that metabolic phenotyping of plasma can be used to screen for alterations in GABA metabolism due to disease or as a consequence of treatment.

## Data Availability

Genomic variant data generated for this study can be found at ClinVar, SUB5205997.

## Ethics Statement

All procedures were performed in accordance with the ethical standards of the United States Department of Health and Human Services and were approved by the Baylor College of Medicine and Washington University Institutional Review Boards. All subjects gave written informed consent in accordance with the Declaration of Helsinki.

## Author Contributions

AK, KP, LE, TD, and SE conceived the ideas and made initial discoveries. AK, KP, and SE drafted and wrote the initial manuscript. LE, MD, MS, TP, and SL recruited patients and obtained clinical data and wrote clinical summaries. LE, QS, VS, SL, AE, TP, WC, AK, KP, and SE reviewed all clinical and biochemical data and edited the manuscript.

## Conflict of Interest

AK, AE, and KP are employees of Metabolon, Inc., and as such, have affiliations with or financial involvement with Metabolon, Inc. QS, LE, WC, VS, SL, and SE are employees of Baylor College of Medicine, which has a partnership with Baylor Genetics that derives revenue from clinical testing. TD is an employee of PerkinElmer Genetics, which derives revenue from clinical testing.

The remaining authors declare that the research was conducted in the absence of any commercial or financial relationships that could be construed as a potential conflict of interest.

## Supplementary Material

DATA SHEET S1 Plasma metabolomic data are provided for all plasma samples obtained and analyzed for this study. Z-scores are shown.

DATA SHEET S2 Data for Figure 4 are provided.

## Abbreviations

ABAT: aminobutyrate aminotransferase
CSF: cerebrospinal fluid
GABA: gamma-amino butyric acid
GDH: glutamate dehydrogenase
IEM: inborn error of metabolism
LC: liquid chromatography
MS: mass spectrometry
SSADH: succinic semialdehyde dehydrogenase.
