# Corrigendum: 2-Pyrrolidinone and Succinimide as Clinical Screening Biomarkers for GABA-Transaminase Deficiency: Anti-seizure Medications Impact Accurate Diagnosis

**DOI:** 10.3389/fnins.2019.01344

**Published:** 2020-01-29

**Authors:** Adam D. Kennedy, Kirk L. Pappan, Taraka Donti, Mauricio R. Delgado, Marwan Shinawi, Toni S. Pearson, Seema R. Lalani, William J. Craigen, V. Reid Sutton, Anne M. Evans, Qin Sun, Lisa T. Emrick, Sarah H. Elsea

**Affiliations:** ^1^Metabolon, Inc., Morrisville, NC, United States; ^2^Department of Molecular and Human Genetics, Baylor College of Medicine, Houston, TX, United States; ^3^Department of Neurology and Neurotherapeutics, Texas Scottish Rite Hospital for Children, The University of Texas Southwestern Medical Center, Dallas, TX, United States; ^4^Department of Pediatrics, Washington University School of Medicine St. Louis, St. Louis, MO, United States; ^5^Department of Neurology, Washington University School of Medicine St. Louis, St. Louis, MO, United States; ^6^Department of Neurology, Baylor College of Medicine, Houston, TX, United States

**Keywords:** 2-pyrrolidinone, vigabatrin, GABA, neurometabolic, inborn error of metabolism, neurotransmitter, 4-aminobutyrate aminotransferase deficiency, GABA-transaminase deficiency

In the original article, there was a mistake in Figure 1 and Figure 3A as published. “Succinamic acid” was omitted from the pathway in Figure 1 and Figure 3A. The corrected figures and figure legends appear below.

**Figure 1:**
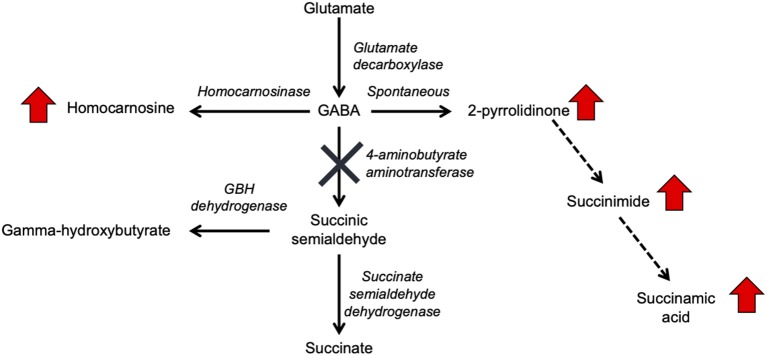
GABA metabolism pathways are altered due to GABA-transaminase deficiency and treatment affecting GABA metabolism. The entire pathway from glutamate conversion to GABA through succinate formation is represented along with the respective enzymes for each step. Due to tissue-specific expression of the enzymes, not all molecules are detected in each biological matrix (e.g., homocarnosine is present below the limit of detection in plasma).

**Figure 3:**
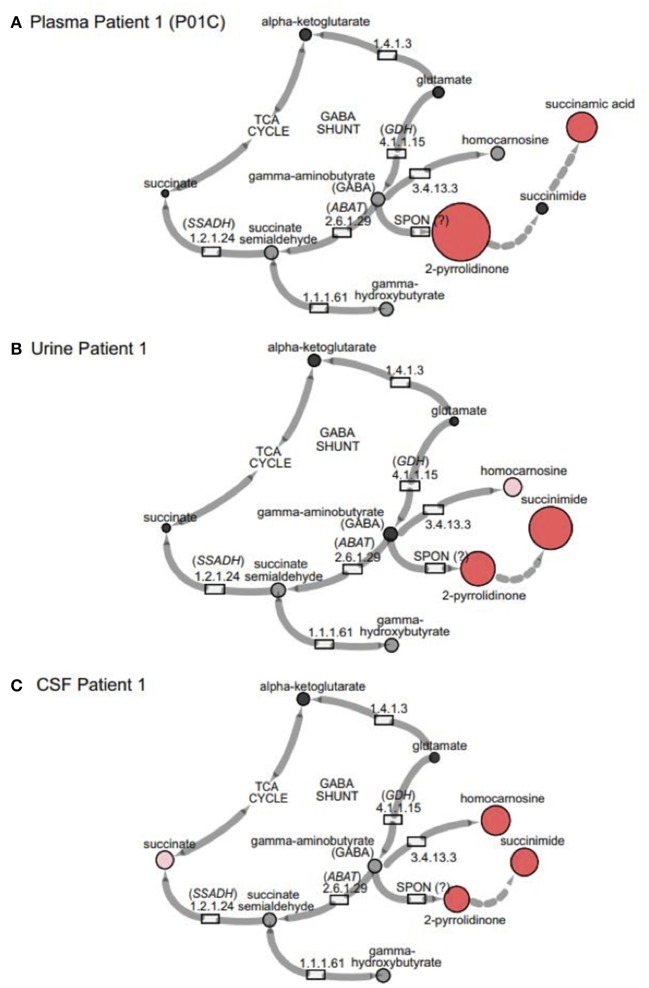
GABA metabolites are altered in GABA-transaminase deficiency and in use of treatments affecting GABA metabolism. Representative pathway images are shown for GABA-transaminase deficiency Patient 1 in **(A)** EDTA Plasma, **(B)** Urine, and **(C)** CSF. Each image shows the relative accumulation of biochemicals (red circles) or trending increases (pink circles, 1.5 ≤ *Z* < 2). The size of each of the circles is representative of the *Z*-score for that biochemical. Black circles represent molecules with *Z*-scores between −1.5 and 1.5 (−1.5 < *Z* < 1.5) or detected rare molecules for which a *Z*-score could not be calculated. Gray circles represent biochemicals in the library but not detected in the samples using Cytoscape to delineate biochemical pathways (http://cytoscape.org) (Shannon et al., 2003). All enzymes in the pathway are denoted by their EC designations. *GDH*, glutamate dehydrogenase; *SSADH*, succinic semialdehyde dehydrogenase; *ABAT*, aminobutyrate aminotransferase; SPON, spontaneous.

Additionally, there was a mistake in Figure 4B as published. “Succinimide” was mistakenly used as the primary biomarker in plasma for the original data analysis, but “succinamic acid” is the proper biomarker. The data have been reanalyzed with succinamic acid to reflect this error. The corrected figure and figure legend appears below.

**Figure 4:**
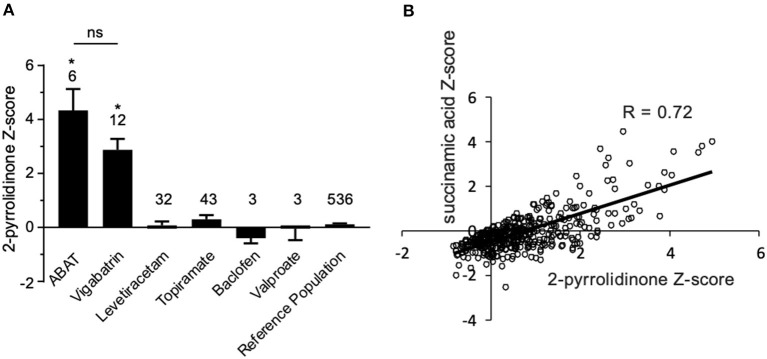
Box plot profile for 2-pyrrolidinone levels detected in plasma for all clinical samples. **(A)** *Z*-scores for all clinical EDTA plasma samples are plotted using a box plot format. ABAT cases show elevated *Z*-scores for 2-pyrrolidinone versus those patients without GABA-transaminase deficiency and treated with vigabatrin, topiramate, and/or valproate. If patients were receiving vigabatrin in addition to other treatments, they were grouped with vigabatrin. Bars represent the mean +/– the standard error of the mean (SEM) of the *Z*-scores for 2-pyrrolidinone for each group. The numbers above the bars represent the number of unique patient samples for each group. ^*^p < 0.05. NS indicates a comparison which was not statically significant, *p* > 0.05. **(B)** Correlation of 2-pyrolidinone levels to succinamic acid levels in clinical plasma samples. Samples which had both 2-pyrrolidinone and succinamic acid *Z*-scored (*n* = 409) are plotted showing a significant positive correlation with these two molecules.

Table 1 and Table 2 have also been updated to reflect these changes:

**Table 1:** Clinical demographics of patients diagnosed with GABA-transaminase deficiency.

**Patient**	**Sample ID^1^^,^^2^**	**Sample type^2^**	Age^*^	**Gender**	**Ethnicity**	***ABAT* variants^+^ clinical report**	**Genome (hg38) Chromosome 16**	**ClinGen canonical ID**	**Medications**	**Diet**
1	*CSF01A*	CSF	15 mon−2 y	Male	Hispanic	c.454C>T (p.Pro152Ser) c.1393G>C (p.Gly465Arg) VUS, *in trans*	g.8764744C>T	CA394688322	Milk of Magnesia, Prevacid, Omega-3, Lansoprazole	Low glutamate
	*P01A*	EDTA Plasma								
	*P01B*	EDTA Plasma								
	*U01A*	Urine					g.8781320G>C	CA394691458		
	**P01C**	**EDTA Plasma**								
2	*U02A* **P02A**	Urine **EDTA Plasma**	6 y	Male	Hispanic	c.631C>T (p.Leu211Phe) homozygous	g.8768220C>T	CA175085	Thiamine, Levocarnitine, Coenzyme Q10, Keppra, Levetiracetam, Clonazepam,	G-button feeds
3	***CSF03A*** **P03A**	**CSF** **EDTA Plasma**	4 y	Male	Caucasian	c.168+1G>A, likely pathogenic variant c.638T>G (p.Phe213Cys), heterozygous VUS, *in trans*	g.8746099G>A g.8768227T>G	CA394692408 CA394688780	No medications	No special diet
4	**P04A** **CSF04A**	**EDTA Plasma** **CSF**	6 y	Female	Caucasian	c.1394G>A (p.Gly465Asp), VUS, homozygous^3^	g.8781321G>A	CA16607451	Miralax, Albuterol, Keppra, Clonazepam	G-tube feeds with Pediasure

1 *Samples in italics were analyzed on Platform version 1. All other samples were analyzed on Platform version 2*.

2 *Succinamic acid was measured on the Polar arm of Platform 2 in samples in boldface*.

3 *Other significant WES variant identified, LRRC7: c.2938C>T (p.R980X), homozygous*.

+ *P80404, ENST00000396600*.

* *y, years; mon, months*.

**Table 2:** Metabolomics identifies altered levels of molecules connected to GABA metabolism in GABA-transaminase deficiency patients^*^.

**Patient**	**Sample**	**Matrix**	**2-pyrrolidinone**	**Succinimide**	**Succinamic Acid^1^**	**Glutamate**	**GABA**	**Succinate**	**Homocarnosine**
1	*CSF01*A	CSF	7.05	5.76	NA	−1.54	0.15	1.65	2.60
	*U01A*	Urine	3.77	4.94	NA	−0.21	0.96	0.21	1.56
	*P01A*	EDTA plasma	6.16	ND	NA	0.71	ND	0.73	ND
	*P01B*	EDTA plasma	6.88	ND	NA	0.86	ND	−0.44	ND
	**P01C**	**EDTA plasma**	4.73	Significant rare^##^	**3.83**	0.03	ND	0.40	ND
2	*U02A*	Urine	0.69	1.55	NA	−0.62	0.92	0.63	0.87
	**P02A**	**EDTA plasma**	1.92	Significant rare^##^	**0.62**	0.92	ND	0.77	ND
3	**P03A**	**EDTA plasma**	2.19	Significant rare^##^	**1.80**	0.57	ND	1.65	ND
	***CSF03A***	**CSF**	5.18	Significant rare^##^	**1.57**	−0.61	0.31	−1.28	1.91
4	**P04A**	**EDTA plasma**	3.58	Significant rare^##^	**2.02**	−0.78	ND	−0.51	ND
	**CSF04A**	**CSF**	Significant rare^#^	Significant rare^##^	**2.25**	−1.15	ND	−1.51	2.75
Non-GABA-T Vigabatrin-treated	***N*** **=** **12**	**EDTA Plasma**	2.88+/−1.41	NA	1.88+/−1.19	0.15+/– 0.90	ND	−1.05+/−2.16	ND

* *Z scores are shown*.

*ND, not detected; NA, not applicable*.

1 *Z-Scores for succinamic acid were calculated for samples in boldface*.

# *Reference ranges were not definable for 2-pyrrolidinone in CSF on platform version 2. 2-pyrollidinone is considered a rare molecule on this platform due to limited detection in the reference population; raw values are considered in the interpretation of this finding*.

## *Reference ranges were not definable for succinimide in EDTA Plasma or CSF on platform version 2. Succinimide is considered a rare molecule on this platform due to limited detection in the reference population; raw values are considered in the interpretation of this finding*.

In line with the changes made above, a correction has also been made to the **Abstract**:

“Broad-scale untargeted biochemical phenotyping is a technology that supplements widely accepted assays, such as organic acid, amino acid, and acylcarnitine analyses typically utilized for the diagnosis of inborn errors of metabolism. In this study, we investigate the analyte changes associated with 4-aminobutyrate aminotransferase (ABAT, GABA transaminase) deficiency and treatments that affect GABA metabolism. GABA-transaminase deficiency is a rare neurodevelopmental and neurometabolic disorder caused by mutations in *ABAT* and resulting in accumulation of GABA in the cerebrospinal fluid (CSF). For that reason, measurement of GABA in CSF is currently the primary approach to diagnosis. GABA-transaminase deficiency results in severe developmental delay with intellectual disability, seizures, and movement disorder, and is often associated with death in childhood. Using an untargeted metabolomics platform, we analyzed EDTA plasma, urine, and CSF specimens from four individuals with GABA-transaminase deficiency to identify biomarkers by comparing the biochemical profile of individual patient samples to a pediatric-centric population cohort. Metabolomic analyses of over 1,000 clinical plasma samples revealed a rich source of biochemical information. Three out of four patients showed significantly elevated levels of the molecule 2-pyrrolidinone (*Z*-score ≥ 2) in plasma, and whole exome sequencing revealed variants of uncertain significance in *ABAT*. Additionally, these same patients also had elevated levels of succinimide or its ring-opened form, succinamic acid, in plasma, urine, and CSF and/or homocarnosine in urine and CSF. In the analysis of clinical EDTA plasma samples, the levels of succinamic acid and 2-pyrrolidinone showed a high level of correlation (*R* = 0.72), indicating impairment in GABA metabolism and further supporting the association with GABA-transaminase deficiency and the pathogenicity of the *ABAT* variants. Further analysis of metabolomic data across our patient population revealed the association of elevated levels of 2-pyrrolidinone with administration of vigabatrin, a commonly used anti-seizure medication and a known inhibitor of GABA-transaminase. These data indicate that anti-seizure medications may alter the biochemical and metabolomic data, potentially impacting the interpretation and diagnosis for the patient. Further, these data demonstrate the power of combining broad scale genotyping and phenotyping technologies to diagnose inherited neurometabolic disorders and support the use of metabolic phenotyping of plasma to screen for GABA-transaminase deficiency.”

to the **Introduction**, **paragraph two**:

“GABA has a relatively short half-life in plasma, is rapidly absorbed, and has both endocrine and hormonal effects (Abel and McCandless, 1992; Li et al., 2015; Maguire et al., 2015). GABA serves as the primary inhibitory neurotransmitter in the human nervous system, and GABA metabolism to succinic semialdehyde helps to regulate its levels and neurotransmitter activity. The accumulation of GABA, either through enzymatic inactivity of ABAT or medical intervention, can result in elevated levels of 2-pyrrolidinone, due to cyclization of GABA (Callery et al., 1978). Conversely, 2-pyrrolidinone can be converted to GABA when it is administered intravenously (Callery et al., 1979) or orally (Fasolato et al., 1988). 2-pyrrolidinone can be converted to succinimide through a two-step reaction (Bandle et al., 1984), and hydrolytic ring opening of cyclic imides such as succinimide can occur through enzymatic (Maguire and Dudley, 1978) and non-enzymatic (Kurono et al., 2008; Lerner et al., 2013) routes.”

and to the **Results**, **paragraph three**:

“By the time additional samples from subsequent ABAT patients were acquired, the configuration of the metabolomics platform had matured. The description of the platform configuration is outlined in the Materials and Methods section, and Table 1 delineates which samples were run on the respective configurations of the platform. Four plasma samples, one from each of the four patients, were subsequently analyzed. 2-pyrrolidinone *Z-*scores ranged from 1.92 to 4.73 across these four EDTA plasma samples (Table 2). For the EDTA plasma sample from Patient 1, succinamic acid, or ring-opened succinimide, showed a relative elevation (*Z* = 3.83), and *Z*-scores for succinamic acid in EDTA plasma and CSF samples from the other GABA-transaminase deficiency patients ranged from 0.62 to 2.25 (Table 2). Succinimide was detected in all EDTA plasma and CSF samples from GABA-transaminase deficiency patients analyzed on platform 2 but was not detected in enough of the healthy reference control population samples to permit the calculation of *Z*-score reference ranges. Of all clinical EDTA plasma samples run on platform version 2, *Z*-scores were obtained for both succinamic acid and 2-pyrrolidinone in 409 samples. Correlation of the values within these samples for succinamic acid and 2-pyrrolidinone showed a high degree of correlation (*R* = 0.72, Figure 4B). The considerable correlation between 2-pyrrolidinone and succinamic acid is suggestive of a product-substrate relationship, even though the biological mechanism leading to the conversion of one to the other is only partially understood.”

Lastly, an author name was incorrectly spelled as “William E. Craigen”. The correct spelling is “William J. Craigen”.

The authors apologize for these errors and state that they do not change the scientific conclusions of the article in any way. The original article has been updated.
